# A Stepwise Telemedicine Follow-Up System for Blood Donors in a University Blood Bank: Prospective Implementation Study

**DOI:** 10.2196/95132

**Published:** 2026-07-31

**Authors:** Ronnarit Boonyarat, Poonsup Sripara, Namrin Boonmawongsa, Boonsong Benjangkaprasert, Weerasak Mahama

**Affiliations:** 1 Blood Transfusion Center Faculty of Medicine Khon Kaen University Mueng Khon Kaen, Khon Kaen Thailand

**Keywords:** mobile health, mHealth, health information technology, process evaluation, telemedicine, blood donor

## Abstract

**Background:**

Conventional mail-based follow-up for blood donors with abnormal postdonation results provides no delivery confirmation and precludes 2-way communication, creating an accountability gap in donor safety.

**Objective:**

This study aimed to implement and evaluate a stepwise telemedicine-based follow-up system for comprehensive blood donor follow-up, including follow-up for abnormal laboratory results, low hemoglobin, and adverse donation reactions, and to measure system coverage, case completion time, and donor satisfaction.

**Methods:**

In this prospective implementation study conducted at Khon Kaen University Hospital from June 20, 2024, to May 30, 2025, all 168 blood donors requiring postdonation were followed up with a stepwise protocol: first, a messaging service (LINE Official Account) was used for initial contact and appointment scheduling, followed by a 2-factor authenticated consultation platform (Dietz) for physician consultation, then telephone, and finally conventional mail. A purpose-developed 15-item satisfaction questionnaire with demonstrated content validity (content validity index=1.00), internal consistency, and a confirmed 5-factor structure was distributed anonymously to all donors enrolled via telemedicine. Case completion time was recorded for all 146 cases with available time-log data.

**Results:**

Of the 168 eligible donors, 146 (86.9%) were enrolled via telemedicine, 16 (9.5%) by telephone, and 6 (3.6%) by mail. The confirmed-contact rate (telemedicine plus telephone) was 96.4% (162/168). Because the prior mail-only system had no mechanism to confirm contact, a comparable rate was not available. The questionnaire response rate was 83.6% (122/146). The median case completion time was 1.90 (IQR 1.42-2.50) hours. Overall satisfaction was 4.44 (SD 0.27) on a 5-point scale. The dimension-level Cronbach α values ranged from 0.886 to 0.987. Completion time did not differ by sex, age, or follow-up reason (all *P*>.05).

**Conclusions:**

The stepwise telemedicine protocol demonstrated high system coverage, rapid case completion, and high donor satisfaction. A residual digital divide (6/168, 3.6% of donors were unreachable by digital means) underscores the need for hybrid follow-up strategies. These findings support broader implementation in blood banking services.

## Introduction

Postdonation follow-up is a cornerstone of blood donor safety. Donors with abnormal postdonation results—including hemoglobin below the threshold, abnormal laboratory findings, or adverse donation reactions—require timely notification and counseling to protect their health and maintain blood supply integrity [1,2]. Failure to complete this process exposes donors to unaddressed health risks and undermines transfusion service accountability [3].

Conventional mail-based notification, common in resource-limited settings, is inherently limited: it provides no delivery confirmation, precludes 2-way communication, and causes delays that may compromise timely clinical intervention [4,5]. At our institution, all preimplementation notifications were dispatched by letter, with no system to verify receipt; therefore, delivery could not be confirmed for any donor. Notably, donor health follow-up and notification remain significant concerns even in well-resourced blood services. In the Netherlands, a nationwide cluster randomized trial demonstrated that standard hemoglobin-based monitoring alone was insufficient to protect whole blood donors from iron deficiency, underscoring the continued need for active donor health follow-up [6]. In Canada, donors with confirmed transfusion-transmissible infections were notified by registered mail over a 17-year period, yet only one-third could later be reached for a follow-up interview, and their recall of the results was often incomplete [7]. These reports indicate that the limitations of 1-way, mail-based communication—namely, no delivery confirmation and no opportunity for 2-way clarification—are not unique to resource-limited settings.

Telemedicine platforms combining asynchronous messaging and synchronous video consultation have demonstrated effectiveness in improving patient engagement and continuity of care across clinical domains [8-10]. In Thailand, LINE—the dominant mobile messaging platform with more than 54 million registered users [11]—has been widely adopted by health care organizations as a LINE Official Account (LINE OA) channel for appointment scheduling and clinical notifications [12]. For secure clinical consultations requiring a higher level of confidentiality, dedicated telemedicine applications provide encrypted 2-way communication with patient identity verification. However, systematic evidence from blood banking settings—particularly in Southeast Asia—remains scarce. This study aimed to (1) implement and evaluate a stepwise telemedicine-based follow-up protocol using a messaging service and a secure consultation platform, (2) assess system coverage and case completion time, and (3) measure donor satisfaction.

More comprehensive follow-up may help donors with abnormal laboratory results or low hemoglobin receive advice, repeat testing, referral, or preventive care sooner. However, this study did not directly evaluate health outcomes.

## Methods

### Study Design and Setting

This was a prospective implementation study (service evaluation) conducted at the Blood Bank, Faculty of Medicine, Khon Kaen University, Khon Kaen, Thailand, between June 20, 2024, and May 30, 2025. The telemedicine system had been implemented approximately 1 year before the evaluation period. Early routine service experience, including operational advantages and problems, motivated this evaluation. Prior to this implementation, the sole donor follow-up modality at this center was postal mail notification; no delivery confirmation mechanism existed, no institutional records of donor acknowledgment or response were maintained, and no returned-mail tracking system was in place. Accordingly, no confirmed-contact rate could be determined under the prior system, as no mechanism existed to verify receipt. The study was conducted in accordance with the Strengthening the Reporting of Observational Studies in Epidemiology (STROBE) reporting guidelines for observational studies [13].

### Ethical Considerations

The study protocol was reviewed by the Center for Ethics in Human Research, Khon Kaen University, which granted an exemption determination (HE671351) under Khon Kaen University Announcement No 2178/2563, category 6.2 (research involving surveys, interviews, and observation), acknowledged at committee meeting 28/2567 (agenda item 3.3.02) on June 19, 2024. The donor follow-up itself was part of routine clinical service; this evaluation analyzed the resulting operational records under the abovementioned exemption. The satisfaction questionnaire was administered only to donors enrolled via telemedicine, who received a participant information sheet describing the survey’s voluntary and anonymous nature; return of the completed questionnaire was taken as implied consent. Donors followed up by telephone or mail received the same clinical service and were not surveyed.

### Telemedicine System Components

**Figure 1:**
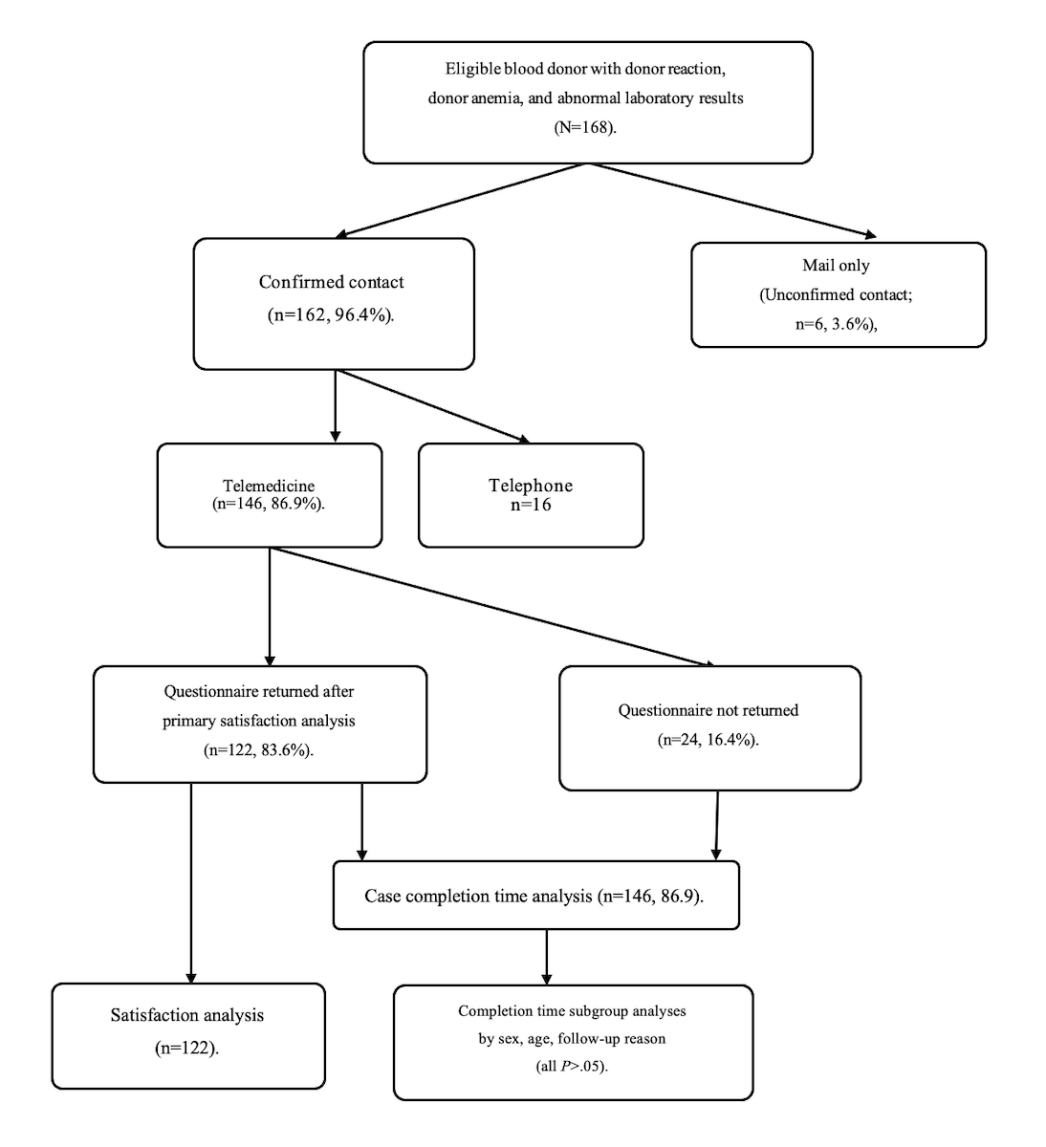
Participant flow diagram. Of 168 eligible blood donors identified during the study period (June 20, 2024, to May 30, 2025), 146 (86.9%) were enrolled via the telemedicine system. A further 16 (9.5%) donors were contacted by telephone, and 6 (3.6%) by conventional mail. Confirmed contact (telemedicine plus telephone) was achieved with 162 (96.4%) of 168 donors. The questionnaire response rate among donors enrolled via telemedicine was 83.6% (122/146).

The institutional telemedicine system comprised 2 integrated components deployed in sequence according to the clinical purpose of each interaction (Figure 1).

The messaging service (LINE OA; LINE Corp; hereafter referred to as the messaging service) served as the primary asynchronous channel for (1) appointment scheduling for clinical consultation and (2) delivery of nonsensitive follow-up messages. The messaging service is integrated into Thailand’s national health care digital infrastructure and allows institutions to broadcast and receive messages through donors’ existing personal accounts, requiring no additional application installation. Rich messaging features (text, images, and PDF attachments) support the transmission of result summaries and health education materials.

The consultation platform (Dietz, Dietz Co Ltd; hereafter referred to as the consultation platform) was used exclusively for direct physician-donor consultations via encrypted audio or video calls. It provides enhanced data privacy through 2-factor authentication (2FA), end-to-end encrypted audio or video transmission, and access controls compliant with the Thai Personal Data Protection Act (PDPA, BE 2562).

The stepwise workflow proceeded as follows: upon identification of an abnormal postdonation result, Blood Bank staff sent an initial notification via the messaging service. An appointment link was dispatched via the messaging service, and the consultation was conducted through the consultation platform. Case completion was defined as confirmed donor acknowledgment via the messaging service and completion of the physician consultation with a documented clinical note. A case in which the donor had acknowledged contact and a consultation had been scheduled but not yet completed was classified as ongoing—not complete—and the case-closure time stamp was recorded only once the consultation had taken place. All 146 telemedicine cases included a physician consultation conducted directly by a physician.

All blood donors aged ≥18 years identified as requiring postdonation follow-up during the study period were eligible and were enrolled as a complete (census) sample. A stepwise protocol was applied: donors were first contacted via the institutional telemedicine system (the messaging service for initial contact and appointment scheduling and the consultation platform for physician consultation). Those unreachable digitally within 24 hours were contacted by telephone; those who remained unreachable received conventional mail. Confirmed contact was defined a priori as receipt of an active donor reply via the messaging service (a text or sticker message; automated read receipts alone were not counted) or a verbal acknowledgment recorded during telephone contact by Blood Bank staff.

### Outcome Measures

The primary outcome was donor satisfaction with the telemedicine follow-up, assessed by a purpose-developed 15-item questionnaire with demonstrated content validity, internal consistency, and a confirmed 5-factor structure. Secondary outcomes were (1) system coverage by channel; (2) case completion time, defined as the interval from the time stamp of the first message dispatched via the messaging service by Blood Bank staff (system-generated log) to the time stamp of case-closure annotation in the Blood Bank information system—comprising confirmed donor acknowledgment and, where applicable, completion of the physician consultation via the consultation platform with a documented clinical note; and (3) factors associated with completion time.

### Satisfaction Questionnaire

A 15-item instrument was developed across 5 dimensions: convenience (Q1-3), privacy and security (Q4-6), clarity of communication (Q7-9), prompt response (Q10-12), and anxiety relief (Q13-15), rated on a 5-point Likert scale. The full instrument is provided in Multimedia Appendix 1.

Content validity was assessed by 5 domain experts (2 transfusion medicine physicians, 1 health informatics physician, 1 clinical pathologist, and 1 senior donor staff member with >10 years’ experience). Each expert rated the relevance of every item on a 4-point scale. From these ratings, we computed, for each item, the item-level content validity index (I-CVI; the proportion of experts rating the item as relevant, ie, ratings of 3 or 4); for the whole instrument, the scale-level content validity index averaged across items (S-CVI/Ave; the mean of the item-level indices); and a chance-corrected agreement statistic, the modified kappa (κ*), which adjusts the I-CVI for agreement expected by chance [14,15]. These indices range from 0 to 1, with I-CVI ≥0.78, S-CVI/Ave ≥0.90, and κ* ≥0.74 conventionally considered acceptable. In our panel, all 15 items achieved I-CVI=1.00, S-CVI/Ave=1.00, and κ*=1.00, indicating that all 5 experts judged every item to be relevant. The panel was composed of clinicians and Blood Bank staff to prioritize the clinical and technical accuracy of the follow-up content; the senior donor-care staff member (>10 years’ experience) contributed a donor-facing operational perspective, and all items were phrased in lay, donor-facing language to support donor comprehension. The questionnaire was distributed anonymously to all donors enrolled via telemedicine. The questionnaire included a brief demographic section (sex, age band, and follow-up reason) but no personal identifiers, and responses were not linked to individual donor records. Respondent demographics were therefore derived from the returned questionnaires.

### Sample Size

The study size was determined by the defined implementation evaluation period from June 20, 2024, to May 30, 2025, rather than by a formal a priori sample size calculation. All eligible donors requiring postdonation follow-up during this period were included in the operational cohort. All 146 (86.9%) donors who received telemedicine follow-up were invited to complete the voluntary satisfaction questionnaire, and 122 (83.6%) completed questionnaires were returned and included in the satisfaction analysis.

### Statistical Analysis

Continuous nonnormal variables are presented as median (IQR), and satisfaction scores are presented as mean (SD). Normality was assessed using the Shapiro-Wilk test. Because the Shapiro-Wilk test indicated nonnormal distributions (*P*<.001), nonparametric tests were used as the appropriate choice for these data. To complement them, we computed bootstrap 95% CIs (10,000 resamples) for the overall satisfaction mean and the median completion time and reported effect sizes (rank-biserial *r* and ε^2^). Precision of the primary satisfaction estimate was assessed using its 95% CI. Between-group comparisons of completion time used the Mann-Whitney *U* test (sex) and the Kruskal-Wallis test (age group and follow-up reason). Because all telemedicine cases included a physician consultation, no with-or-without-consultation comparison subgroup existed for this analysis. Internal consistency was assessed by Cronbach α (acceptable threshold: ≥0.70) [16]. The 25-hour threshold was defined based on the workflow’s expected 24-hour completion window; values exceeding 25 hours were considered operational outliers. A sensitivity analysis excluded cases with operationally explained outlier completion times (>25 hours). All statistical analyses were performed using R (version 4.3.3; R Foundation for Statistical Computing). The following packages were used: *stats* (version 4.3.3; for the Shapiro-Wilk normality test, Mann-Whitney *U* or Wilcoxon rank-sum test, and Kruskal-Wallis test), *psych* (version 2.6.1; for Cronbach α computation), *ggplot2* (version 4.0.2), *dplyr* (version 1.1.4), *patchwork* (version 1.3.2), and *cowplot* (version 1.2.0) for visualization. Statistical significance was defined as *P*<.05 (2-tailed). Exploratory factor analysis was conducted as a preliminary examination of the instrument’s dimensional structure. Detailed methods and results, including factor loadings and the scree plot, are provided in Multimedia Appendix 2.

## Results

### System Coverage and Participant Flow

During the 11-month study period, 168 (0.6%) of 30,018 donors were identified as requiring postdonation follow-up, and all were enrolled. Of these 168 donors, 146 (86.9%) were enrolled via telemedicine, 16 (9.5%) by telephone, and 6 (3.6%) by mail only (Figure 1). Confirmed contact (defined in the Methods section) was achieved in 162 (96.4%) of 168 donors. The 6 (3.6%) donors reached by mail only represent cases with unconfirmed delivery. Of the 146 donors enrolled via telemedicine, 122 (83.6%) returned completed questionnaires. Full demographic profiles were available for all 22 (13.1%) donors followed up by telephone (n=16, 9.5%) or mail (n=6, 3.6%).

### Participant Characteristics

The 122 (83.6%) telemedicine questionnaire respondents comprised 65 (53.3%) male donors and 57 (46.7%) female donors. The most common age group was 18 to 25 years (33, 27%), followed by 26 to 35 years (28, 23%). Follow-up reasons were abnormal laboratory results (46, 37.7%), low hemoglobin (45, 36.9%), and adverse donation reactions (31, 25.4%). Demographic data for the 24 (16.4%) nonrespondents could not be obtained, as the anonymous design prevented linkage to individual donors.

**Figure 2:**
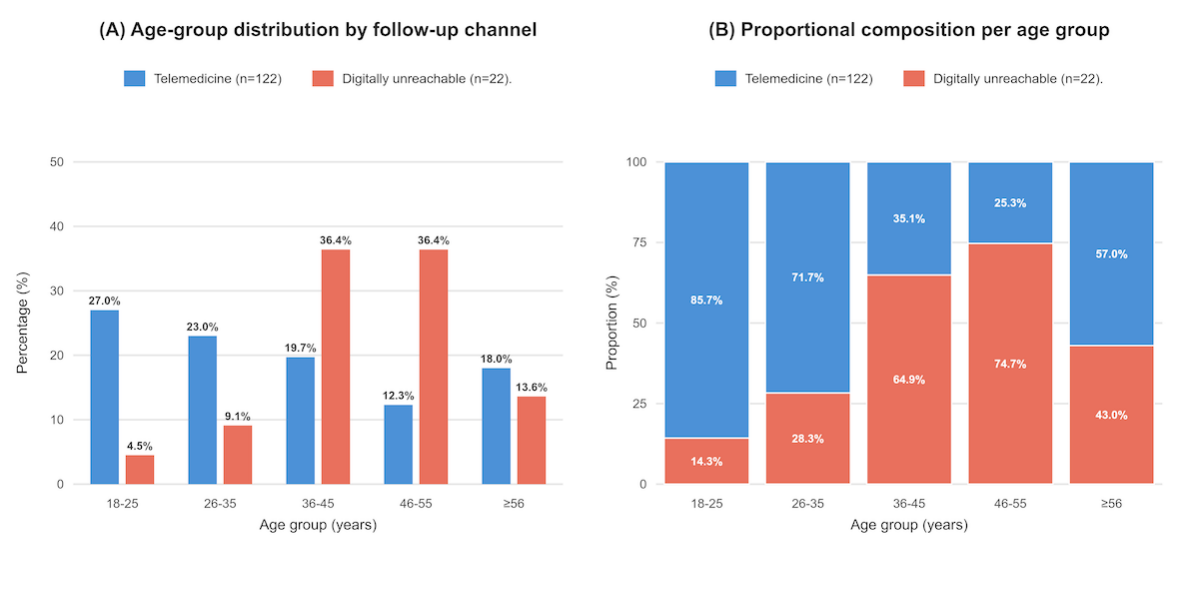
Age group distribution of telemedicine questionnaire respondents (n=122) vs digitally unreachable donors (n=22). Bar charts displaying (A) the percentage distribution of each donor group by age category (18-25, 26-35, 36-45, 46-55, and ≥56 years) and (B) proportional distribution of donors by age category within each study group. Data are presented as descriptive proportions only; no inferential comparison between groups was made. The 36- to 55-year age cohort constituted 72.7% (16/22) of digitally unreachable donors and 32% (39/122) of telemedicine respondents. Figures were generated using ggplot2 (version 4.0.2) and patchwork (version 1.3.2) in R (version 4.3.3).

Demographic profiles were available for all 22 (13.1%) digitally unreachable donors (from operational contact records; Table 1). Female donors predominated (14/22, 63.6%). Descriptively, the 36- to 55-year age group constituted 72.7% (16/22) of digitally unreachable donors, compared with 32% (39/122) of donors enrolled via telemedicine (Figure 2). As the digitally unreachable group represents donors for whom telemedicine contact was attempted but unsuccessful—not an independently sampled group—these figures are presented as descriptive profiles of digital accessibility rather than inferential comparisons between groups.

**Table 1:** Demographic profiles of donors enrolled via telemedicine vs digitally unreachable donors (descriptive)^a^.

Characteristics			Donors enrolled via telemedicine (n=122), n (%)		Digitally unreachable donors (n=22), n (%)
**Sex**					
	Male	65 (53.3)		8 (36.4)	
	Female	57 (46.7)		14 (63.6)	
**Age group (years)**					
	18-25	33 (27)		1 (4.5)	
	26-35	28 (23)		2 (9.1)	
	36-45	24 (19.7)		8 (36.4)	
	46-55	15 (12.3)		8 (36.4)	
	≥56	22 (18)		3 (13.6)	
**Follow-up reason**					
	Abnormal laboratory results	46 (37.7)		9 (40.9)	
	Low hemoglobin	45 (36.9)		6 (27.3)	
	Adverse donation reactions	31 (25.4)		7 (31.8)	

^a^The digitally unreachable group comprised donors for whom telemedicine contact was attempted but unsuccessful. These donors do not represent an independently sampled comparison group. All values are presented as descriptive proportions.

### Case Completion Time

All 146 telemedicine cases reached completion during the study period. Time-log data were available for all 146 cases. All 146 telemedicine cases included a physician consultation conducted directly by a physician; consequently, no with-or-without-consultation comparison subgroup was available for completion time analysis. The median completion time was 1.90 (IQR 1.42-2.50; 95% CI 1.80-2.10; range 0.50-42.7) hours. A total of 4 (2.7%) cases exceeded the prespecified 25-hour threshold (range 27.5-42.7 hours): 2 (50%) due to donor nonresponse via the messaging service and 2 (50%) due to late-week repeat laboratory requests. In the sensitivity analysis excluding these 4 (2.7%) cases (142/146, 97.3%), the median completion time was 1.90 (IQR 1.40-2.48) hours, consistent with the primary analysis. Completion time did not differ significantly by sex (Mann-Whitney *U*, *W*=2587.5; *P*=.79; rank-biserial, *r*=0.03), age group (Kruskal-Wallis, H_4_=1.97; *P*=.74; ε^2^<0.01), or follow-up reason (Kruskal-Wallis, H_2_=0.53; *P*=.77; ε^2^<0.01). All effect sizes indicated negligible between-group differences (Table 2 and Table 3).

**Table 2:** Case completion time (N=146)^a^.

Statistics	All cases	Excluding outliers (n=142)
Case completion time (h), median (IQR)	1.90 (1.42-2.50)	1.90 (1.40-2.48)
Case completion time (min), median (IQR)	114 (85-150)	114 (84-149)
Case completion time (h), mean (SD; range)	2.78 (5.26; 0.50-42.7)	1.93 (0.74; 0.50-3.90)

^a^Outliers comprised 4 cases with completion times >25 hours (2 due to donor nonresponse and 2 due to operational laboratory delays).

**Table 3:** Subgroup comparisons.^a^

Characteristics	Test statistic (*W* or *H*), effect size (r or ε^2^)	*P* value	Excluding outliers (n=142)
Sex (Mann-Whitney *U* test)	*W*=2587.5, *r*=0.03	.79	—^b^
Age group (Kruskal-Wallis test)	*H*_4_=1.97, ε^2^<0.01	.74	—
Follow-up reason (Kruskal-Wallis test)	*H*_2_=0.53, ε^2^<0.01	.77	—

^a^Subgroup comparisons (sex, age group, follow-up reason) were performed on the primary case set (n=146) only.

^b^Not applicable (subgroup comparisons were not repeated in the sensitivity analysis excluding outliers (n=142).

### Donor Satisfaction and Internal Consistency

Of the 146 donors enrolled via telemedicine and invited to complete the questionnaire, 122 returned completed questionnaires, corresponding to a response rate of 83.6%. The mean overall satisfaction score was 4.44 (SD 0.27; 95% CI 4.40-4.49; Table 4; [17]). Dimension scores ranged from 3.86 (SD 1.05; convenience) to 4.62 (SD 0.44; prompt response). Dimensions 2 to 5 demonstrated ceiling effects (mean ≥4.56, SD ≤0.47). Dimension-level Cronbach α ranged from 0.886 to 0.987 (indicating good-to-excellent internal consistency across all dimensions), with all within-dimension interitem correlations between *r*=0.687 and *r*=0.971. Overall Cronbach α was 0.665, reflecting scale heterogeneity between dimension 1 (SD~1.05) and dimensions 2 to 5 (SD~0.45) rather than poor item quality.

Exploratory factor analysis results are provided in Multimedia Appendix 2.

**Table 4:** Donor satisfaction by dimension and overall (n=122)^a^.

Dimensions	Items	Mean (SD)	Median (IQR)	Cronbach α	Interpretation
Dimension 1: convenience	1-3	3.86 (1.05)	4.00 (3.00-5.00)	0.987	Excellent
Dimension 2: privacy and security	4-6	4.56 (0.45)	4.67 (4.00-5.00)	0.893	Good
Dimension 3: clarity of communication	7-9	4.57 (0.47)	5.00 (4.00-5.00)	0.938	Excellent
Dimension 4: prompt response	10-12	4.62 (0.44)	5.00 (4.00-5.00)	0.886	Good
Dimension 5: anxiety relief	13-15	4.59 (0.46)	5.00 (4.00-5.00)	0.922	Excellent
Overall	1-15	4.44 (0.27)	4.47 (4.22-4.60)	0.665^b^	Questionable

^a^Interpretation of Cronbach α follows the study by George and Mallery [17]: ≥0.9=“excellent,” ≥0.8=“good,” ≥0.7=“acceptable,” and ≥0.6=“questionable.”

^b^The overall Cronbach α reflects scale heterogeneity (dimension 1: SD~1.05 vs dimensions 2 to 5: SD~0.45); therefore, the dimension-level Cronbach α values are considered the primary reliability indicator.

## Discussion

### Principal Findings

This implementation study demonstrated that a stepwise telemedicine-based follow-up system achieved a confirmed-contact rate of 96.4% (162/168). Because the prior mail-only system had no mechanism to verify receipt, a comparable preimplementation rate is not available; therefore, we frame this not as an increase from a measured baseline but as the introduction of a confirmable, 2-way follow-up pathway where none previously existed. The resulting gain in donor safety accountability reflects the responsibility assumed by the blood establishment and the response options now available to donors—the capacity to confirm contact and to reply—rather than a numerical change in a previously unmeasurable rate.

Prior reports of conventional donor notification illustrate the limitations of 1-way channels. Arthi et al [4] found that among 117 seropositive blood donors notified by conventional channels, only 70% responded and 30% remained unreachable owing to inaccurate contact information. Similarly, Mittal et al [3] showed that adding active telephone calls to letters improved donor return. These studies differ from ours in donor population, setting, and outcome definition, so their findings are not directly comparable to our 96.4% (162/168) confirmed-contact rate; we cite them as context for the recognized shortcomings of mail-only notification rather than as a numerical benchmark. The widespread adoption of LINE across demographic groups in Thailand, coupled with validated digital messaging platforms for postprocedure clinical follow-up [12], likely contributed to this high uptake. The seamless handoff from the messaging service to the consultation platform—with clinical-grade confidentiality through 2FA—minimized technical friction while ensuring PDPA compliance [18].

### Comparison With Prior Work

The stepwise design was critical to achieving high population coverage. Telemedicine alone enrolled 146 (86.9%) of 168 eligible donors; the addition of telephone contact increased confirmed communication to 96.4% (162/168). This layered, channel-redundant architecture reflects the principle that sustainable telehealth implementation requires flexible fallback mechanisms to serve populations with variable digital access—a requirement articulated by Thomas et al [18] in their framework for sustaining telehealth beyond the COVID-19 pandemic, which emphasized that training, funding, and multichannel capability are prerequisites for equitable uptake. The 6 (3.6%) donors reachable only by mail represent a residual high-risk subgroup for whom follow-up completion cannot be confirmed; proactive identification of digitally unreachable donors at blood donation registration should be a priority in future implementation.

Descriptive profiling of the 22 (13.1%) digitally unreachable donors reveals a pattern that warrants discussion. The 36- to 55-year age group constituted 72.7% (16/22) of this subgroup, compared with 32% (39/122) of donors enrolled via telemedicine, whereas donors aged ≥56 years were proportionally well represented among telemedicine users (22/122, 18% vs 3/22, 13.6%). It is important to note that the digitally unreachable group is not an independently sampled comparison group—these donors represent those for whom telemedicine contact was attempted but unsuccessful—and its small size (22/168, 13.1%) precludes formal inferential testing. Nevertheless, the descriptive pattern is consistent with evidence from multistakeholder analyses of digital health equity, which caution that age alone is an oversimplified proxy for digital exclusion and that occupational and social factors are often more proximate determinants [19]. In the Thai context, the messaging service is widely used across age groups, including by older adults for family communication [20], which may explain the relatively high telemedicine uptake among donors aged ≥56 years. We do not advance a specific explanation for the higher proportion of digitally unreachable donors in the 36- to 55-year age group; this subgroup was small (22/168, 13.1%), was not independently sampled, and the available data could not identify the underlying reasons. We note only that occupational and social factors are recognized to influence digital health engagement independently of age [21] and that future registration workflows should capture donors’ preferred personal digital contact channels.

Rapid case completion supports the operational feasibility of the telemedicine workflow. The identifiable causes of delayed cases suggest practical opportunities for improvement, including automated reminder protocols and review of late-week laboratory scheduling.

High satisfaction supports the acceptability of the telemedicine service. The comparatively lower convenience score suggests that initial onboarding and appointment scheduling may remain barriers for some donors and should be prioritized in future service improvement. The ceiling effects and limitations of the instrument are discussed in the next section.

### Limitations

This study has several limitations. First, it was a single-center implementation study without a concurrent control group. The comparison with the prior mail-only system is therefore a historical, uncontrolled comparison susceptible to secular trends and confounding (eg, changes in staffing, donor population, or institutional practice over time). Moreover, because the prior system had no mechanism to confirm contact, the preimplementation rate is not available rather than zero. Therefore, we make no causal claim that the telemedicine system itself produced the observed confirmed-contact rate, and the before-after comparison should be interpreted with caution.

Second, anonymous administration of the questionnaire, while protecting donor privacy, precluded any assessment of nonresponse bias: 24 (16.4%) of 146 donors enrolled via telemedicine did not return a questionnaire, and neither their characteristics nor their satisfaction could be compared with those of respondents. This is a nontrivial fraction for a sample of this size. Because less-satisfied donors may be less likely to respond, the satisfaction estimates may be biased upward and should be regarded as an upper bound rather than an unbiased estimate; we cannot exclude meaningful nonresponse bias. As a proxy indicator, the median case completion time for the full telemedicine cohort (n=146; median 1.90, IQR 1.42-2.50 hours) was identical to that of the questionnaire respondent subgroup, and the 24 nonrespondents did not cluster among the 4 outlier cases, providing limited but partial reassurance regarding severe nonresponse bias.

Third, satisfaction was assessed only among donors who used the telemedicine system; those followed up by telephone or mail were not surveyed. The satisfaction sample is therefore a self-selected, digitally engaged subgroup, introducing selection bias that likely favors more positive evaluations and limits generalizability to all donors requiring follow-up, while also precluding cross-channel comparison.

Fourth, the content validity panel comprised clinicians and staff without donor or patient representation. Although items were drafted in donor-facing language, this composition may not fully capture the lived donor experience that the instrument is intended to measure, and future work should incorporate donor cognitive interviewing or pilot testing to strengthen content validity from the user perspective.

Fifth, 4 of the 5 satisfaction dimensions exhibited ceiling effects, which constrained the instrument’s discriminative capacity and its sensitivity to detect change in future longitudinal use. No formal a priori sample size calculation was used to determine recruitment. Although the achieved questionnaire sample provided a relatively narrow CI for the primary descriptive satisfaction estimate, the study may have had limited statistical power for subgroup comparisons and uncommon outcomes. The sample was also not intended to support broader psychometric validation of the questionnaire. Broader psychometric validation, including convergent or discriminant validity, criterion validity, test-retest reliability, and confirmation in an independent sample, was not conducted. The observed ceiling effects further limit claims about discriminant validity.

Sixth, cost data were not collected, and future implementation studies should document the per-case cost of telemedicine vs conventional notification to strengthen the economic argument for adoption. In addition, the low-prevalence follow-up caseload (168/30,018, 0.6% of donors; refer to the Results section for details) may differ from that of higher-volume centers, which could limit generalizability.

Finally, this evaluation measured operational and experiential outcomes—system coverage, case completion time, and donor satisfaction—but did not assess clinical end points, such as donor adherence to follow-up recommendations (eg, attending recommended repeat testing or physician consultation) or downstream improvements in donor health status (eg, resolution of low hemoglobin). Whether the high reachability and satisfaction observed here translate into better clinical outcomes remains unknown and represents an important objective for future studies. Future multicenter studies with a prospective comparative design and cross-channel satisfaction measurement are warranted.

### Conclusions

A stepwise telemedicine-based follow-up system achieved a confirmed-contact rate of 96.4% (162/168) among blood donors requiring postdonation follow-up, with telemedicine enrolling 86.9% (n=146) and telephone follow-up accounting for a further 9.5% (n=16), whereas confirmation of contact was not previously possible under the mail-only approach. High donor satisfaction, consistent completion times, and satisfactory instrument reliability support both the safety and acceptability of this implementation. A residual digital divide warrants targeted hybrid strategies. These findings provide preliminary evidence supporting implementation and future multicenter evaluation, with potential for policy-level adoption in blood banking services pending successful replication.

## Appendix

Multimedia Appendix 1 Donor Satisfaction Questionnaire.

Multimedia Appendix 2 Exploratory factor analysis of the 15-item donor satisfaction instrument.

## Abbreviations

2FA: 2-factor authentication
I-CVI: item-level content validity index
LINE OA: LINE Official Account
PDPA: Personal Data Protection Act
S-CVI/Ave: scale-level content validity index averaged across items
STROBE: Strengthening the Reporting of Observational Studies in Epidemiology

## Data Availability

The datasets analyzed during this study are available from the corresponding author on reasonable request, subject to applicable ethics and privacy constraints.
