# Supplementary material for: A Stepwise Telemedicine Follow-Up System for Blood Donors in a University Blood Bank: Prospective Implementation Study
Source: JMIR Form Res. 2026 Jul 31;10:e95132. doi: 10.2196/95132 (PMC13476728; doi:10.2196/95132)
Supplement: Supplementary file 1 [file formative_v10i1e95132_app1.docx]

**Multimedia Appendix 1. Donor Satisfaction Questionnaire**

*Satisfaction Questionnaire for Telemedicine-Based Blood Donor Follow-up — Blood Transfusion Center, Faculty of Medicine, Khon Kaen University.*

The instrument comprises 15 items across five dimensions (three items each), each rated on a five-point Likert scale: 1 = very dissatisfied, 2 = dissatisfied, 3 = neutral, 4 = satisfied, 5 = very satisfied. The questionnaire was administered to donors in Thai; the English translation is shown below. The original Thai version is available from the corresponding author on request.

**Demographic Information**

D1. Gender: ☐ Male	☐ Female	☐ Prefer not to say

D2. Age group: ☐ 18–25	☐ 26–35	☐ 36–45	☐ 46–55	☐ >55

D3. Reason for consultation: ☐ Low hemoglobin (Hb)	☐ Infectious disease laboratory result	☐ Donor reaction

|  | **Very dissatisfied (1)** | **Dissatisfied (2)** | **Neutral (3)** | **Satisfied (4)** | **Very satisfied (5)** |
| --- | --- | --- | --- | --- | --- |
| **Dimension 1 — Convenience** |  |  |  |  |  |
| Q1. The telemedicine system has steps that are easy to understand. |  |  |  |  |  |
| Q2. I could access the system conveniently without traveling to the blood bank. |  |  |  |  |  |
| Q3. I was able to complete the follow-up at a time convenient for me. |  |  |  |  |  |
| **Dimension 2 — Privacy and Security** |  |  |  |  |  |
| Q4. I felt that my personal data and test results were kept confidential. |  |  |  |  |  |
| Q5. The telemedicine system provided secure transmission of my health information. |  |  |  |  |  |
| Q6. I felt comfortable discussing my blood test results through this channel. |  |  |  |  |  |
| **Dimension 3 — Clarity of Communication** |  |  |  |  |  |
| Q7. Staff communicated test results and health information clearly and understandably. |  |  |  |  |  |
| Q8. I had sufficient opportunity to ask questions or request additional information. |  |  |  |  |  |
| Q9. The information I received was complete enough to guide my health care. |  |  |  |  |  |
| **Dimension 4 — Prompt Response** |  |  |  |  |  |
| Q10. Staff responded to my appointment or contact in a timely manner. |  |  |  |  |  |
| Q11. I did not wait an unreasonably long time before being contacted by the blood bank. |  |  |  |  |  |
| Q12. The overall speed of the telemedicine follow-up process met my expectations. |  |  |  |  |  |
| **Dimension 5 — Anxiety Relief** |  |  |  |  |  |
| Q13. Receiving my test results through the telemedicine system helped reduce my anxiety. |  |  |  |  |  |
| Q14. I felt that staff were attentive and reassuring during the follow-up. |  |  |  |  |  |
| Q15. Overall, I felt more at ease and reassured after receiving follow-up through telemedicine. |  |  |  |  |  |

*Additional comments (open-ended, optional): ____________________________*
