# Supplementary material for: A Stepwise Telemedicine Follow-Up System for Blood Donors in a University Blood Bank: Prospective Implementation Study
Source: JMIR Form Res. 2026 Jul 31;10:e95132. doi: 10.2196/95132 (PMC13476728; doi:10.2196/95132)
Supplement: Supplementary file 2 [file formative_v10i1e95132_app2.docx]

**Multimedia Appendix 2
Exploratory Factor Analysis of the 15-Item Donor Satisfaction Instrument**

# Methods

The dimensional structure of the purpose-developed 15-item satisfaction instrument was examined by exploratory factor analysis using maximum-likelihood extraction with oblimin rotation. Sampling adequacy was assessed using the Kaiser–Meyer–Olkin (KMO) measure and Bartlett’s test of sphericity. The number of factors was examined using the Kaiser criterion (eigenvalue >1) together with the scree plot. The five-factor solution was inspected to determine whether the items loaded onto their a priori dimensions.

# Results

The KMO measure was 0.74, and Bartlett’s test of sphericity was significant (χ²=1682.4, P<.001). Five factors had eigenvalues greater than 1 (3.36, 2.93, 2.75, 2.38, and 1.75), accounting for 81.5% of the cumulative variance. Items loaded strongly on their intended dimensions, with low cross-loadings (Table S1). These findings provide preliminary evidence regarding the instrument’s dimensional structure in this study sample; broader psychometric validation was not conducted.

**Table S1. Exploratory factor loadings of the 15-item donor satisfaction instrument**

| **Item** | **A priori dimension** | **Loading on intended factor** | **Highest cross-loading** |
| --- | --- | --- | --- |
| Q1–Q3 | Convenience | 0.98 / 0.99 / 0.97 | <0.02 |
| Q4–Q6 | Privacy and Security | 0.77 / 0.86 / 0.94 | <0.07 |
| Q7–Q9 | Clarity of Communication | 0.91 / 0.91 / 0.92 | <0.07 |
| Q10–Q12 | Prompt Response | 0.86 / 0.82 / 0.87 | <0.06 |
| Q13–Q15 | Anxiety Relief | 0.89 / 0.92 / 0.87 | <0.07 |

# Figure S1. Scree plot of the 15-item donor satisfaction instrument

*Eigenvalues are plotted against factor number; the dashed line marks the Kaiser criterion (eigenvalue=1). Five factors exceeded 1 before a sharp decline, supporting the five a priori dimensions (KMO=0.74; Bartlett P<.001).*
